# Cases of Tetanus Treated in Home Military Hospitals (from Aug. 1, 1915 to July 31, 1916)

**Published:** 1918-01

**Authors:** 


***Cases of Tetanus Treated in Home Military Hospitals (from Aug. 1, 1915 to July 31, 1916).*** A Second Analysis by Sir David Bruce, Surgeon-General, Army Medical Service. Abs. from the *Lancet*, Dec. 2, 1916.

Statistics for 195 cases of tetanus from war wounds treated in military hospitals in England during the year 1915-16 are compiled with a view to ascertaining information on the disputed problems of the disease. Various diagrams and tables are presented. The author admits that the number of cases was too small and the methods of treatment too varied, to make any conclusion based upon his statistics reliable or final.

He notes especially that cases of a short incubation period (from 4 to 10 days) were most fatal (81 o/o). Those with an incubation period of from 11 to 25 days resulted in a mortality of 52.2 o/o. For cases with a longer incubation period up to 330 days, the mortality was 27.2 o/o. More cases occurred on the 11th day after the wound than on any other.

15 cases are reported in which tetanus followed operations on wounds which had previously shown no symptoms of tetanus, and which had not been treated prophylactically. There were 9 recoveries and 6 deaths. In several cases tetanus set in within 2 days of the operation.

As to the effects of operation after tetanus has set in, the statistics are not conclusive. Of 21 cases thus reported, there were 11 recoveries and 10 deaths. B. finds the statistics especially disappointing as throwing light upon the best method of employing serum. Of the 77 men who were known to have received a prophylactic injection 44 recovered and 33 (42.8 o/o) died. 52 o/o of the remaining 118 died. It is believed that most of these had also received prophylactic injections at the front, although the fact was not reported. There was a slightly lower percentage of mortality among those inoculated on the day of the injury than among those who received injections later. The average incubation period for the fatal cases was somewhat shorter than for those who recovered.

The returns for curative treatment of 175 cases showed a mortality of 47.4 o/o. As to the advantage of intrathecal injections, the statistics are discouraging. Only 7 cases were treated by this method alone. Of these 2 recovered and 5 died. This was a poorer showing than for any other method of treatment. Of the 75 cases in which intrathecal injections were given in addition to others, the mortality was 46.7 o/o. Of 8 cases treated by intramuscular injections alone, there were 7 recoveries. It was observed, however, that when the intrathecal injection was administered on the day of the onset of symptoms, there was a mortality of only 33 o/o out of 18 cases as against 52 o/o and 57 o/o for other treatments. When the treatment was given one or two days later, only a slight difference of from 6 o/o to 9 o/o in favor of this method of administering serum was indicated.

The writer advises the following course of treatment :

" *a*) Place in a quiet, darkened room under the care of sympathetic and capable nurse. Rest, sleep, and food, are looked upon as the first essentials of treatment.

" *b*) If thorough surgical treatment is carried out on wounds from the beginning, so as not to allow the presence of necrotic tissues or foreign bodies, the number of cases of tetanus should sensibly diminish, if not altogether disappear. But if a case does occur, then the wound should not actively be interfered with until the tetanic symptoms have subsided.

" *c*) Intrathecal injections of large doses of antitoxin, of high potency if available, should be begun at once, and supplemented by intramuscular and subcutaneous injections.

" *d*) In addition, if necessary, the patient should receive sedatives, of which morphine in 1/4 gr. doses, administered every 4 hours, is perhaps the most suitable. Chloral, chloretone, and other sedatives are also given by the mouth or rectum. "

